# Bilateral pheochromocytoma with ganglioneuroma component associated with multiple neuroendocrine neoplasia type 2A: a case report

**DOI:** 10.1186/s13256-017-1364-6

**Published:** 2017-08-01

**Authors:** Boubacar Efared, Gabrielle Atsame-Ebang, Soufiane Tahirou, Khalid Mazaz, Nawal Hammas, Hinde El Fatemi, Laila Chbani

**Affiliations:** 1 Department of Pathology, Hassan II University Hospital, Fès, Morocco; 2 Department of Radiology, Hassan II University Hospital, Fès, Morocco; 3 Department of General and Visceral Surgery, Hassan II University Hospital, Fès, Morocco; 4 Faculty of Medicine and Pharmacology, Sidi Mohamed Ben Abdellah University, Fès, Morocco

**Keywords:** Composite pheochromocytoma/paraganglioma, MEN 2, Ganglioneuroma

## Abstract

**Background:**

Composite pheochromocytoma/paragangliomas are very rare tumors composed of ordinary pheochromocytoma paragangliomas associated with neurogenic tumors. Several hereditary susceptibility disorders are known to be associated with pheochromocytoma/paragangliomas such as multiple endocrine neoplasia type 2 (2A or B). To the best of our knowledge, only four cases of composite pheochromocytoma/paragangliomas associated with multiple endocrine neoplasia type 2 have been reported.

**Case presentation:**

A 40-year-old Arabic woman presented with headache, palpitations, paroxysmal hypertension, and weight loss, which she had had for the last 3 years. She had a familial history of diabetes and multiple endocrine neoplasia type 2. A radiological examination revealed thyroid lesions and bilateral adrenal medulla tumors. Our patient had undergone bilateral adrenalectomy, total thyroidectomy with cervical lymphadenectomy, and parathyroidectomy. A pathological examination confirmed the multiple endocrine neoplasia type 2A consisting of left medullary pheochromocytoma, right medullary composite pheochromocytoma-ganglioneuroma, medullary carcinoma of the thyroid with lymph node metastasis and parathyroid hyperplasia. A genetic analysis also revealed that our patient had a *RET* germline mutation.

**Conclusion:**

Composite pheochromocytoma/paraganglioma associated with multiple endocrine neoplasia type 2 is a very rare occurrence, as the current literature provides only a few cases. Further reported cases are needed in order to understand the behavior and the pathogenesis of this uncommon entity.

## Background

Composite pheochromocytoma/paraganglioma (CPC/PG) is a rare entity composed of classic PC/PG associated with other neurogenic tumors such as neuroblastoma, ganglioneuroblastoma, ganglioneuroma (GN), or malignant peripheral nerve sheath tumors (MPNST) [1]. In the literature, ganglioneuroma is the most reported component of composite tumors [2]. The adrenal medulla is the common site of these tumors, however, extramedullary locations (CPG) can be encountered. Cases of CPC/PG have been reported in association with neurofibromatosis (NF), gastrointestinal stromal tumors (GIST), multiple endocrine neoplasia (MEN), and von Hippel-Lindau syndrome (vHL) [2, 3]. The management and the prognosis of CPC/GN seem to be similar to their ordinary-type counterpart (PC/PG), but based on a few reported cases to date, little is known about their real behavior [2, 4, 5]. To the best of our knowledge, only four cases of CPC/PG associated with MEN 2 have been previously reported in the literature [6–9]. The first case was reported by Brady *et al*. in 1997 in a 34-year-old man with MEN 2A. In 1998, Matias-Guiu *et al.* reported another case of CP in a 49-year-old man with MEN 2A. The two reported cases were located in the left adrenal medulla and the neurogenic components were ganglioneuroma and ganglioneuroblastoma, respectively. Cases associated with MEN 2B have also been reported. Charfi *et al*. reported in 2008 the first case of CP associated with MEN type 2B. More recently, in 2016, Yamasaki *et al.* reported a case of a composite retroperitoneal paraganglioma in a 59-year-old man.

We report here an additional case of CPC in a 40-year-old woman with MEN 2A. We discuss the clinicopathological issues and perform a review of the literature in order to improve understanding of this very rare type of tumor.

## Case presentation

**Fig. 1:**
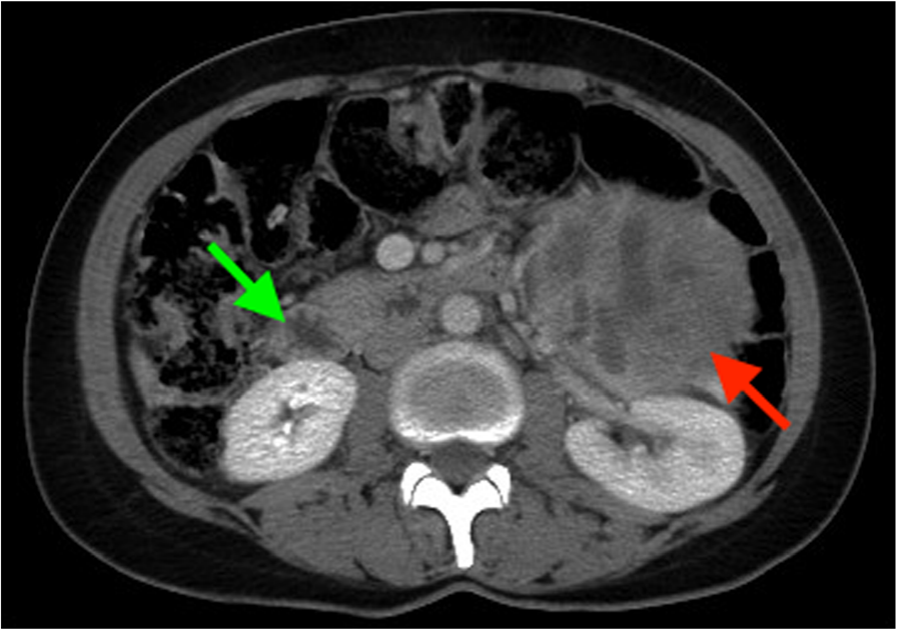
The abdominal computed tomography scan shows a left adrenal mass (*red arrow*) and a well-encapsulated nodule of the right adrenal gland (*green arrow*). The two lesions have central hypodense areas

A 40-year-old Arabic woman presented with headache, palpitations, paroxysmal hypertension and weight loss, which she had had for 3 years. She had a history of treated type 2 diabetes. She had also a familial history of type 2 diabetes and MEN 2A. Her sister had undergone thyroidectomy and adrenalectomy for medullary carcinoma of the thyroid and pheochromocytoma. A physical examination showed an enlarged and firm thyroid mass which moved with swallowing, without cervical adenopathy. Our patient had no hypertension on admission. A cervical ultrasound scan found nodular heterogeneous calcified and cystic lesions of the thyroid gland, the larger lesion was located at the left lobe and measured 1.8 × 1.3 cm. An abdominal computed tomography (CT) scan revealed a left adrenal mass and a well-encapsulated nodule of the right adrenal gland (Fig. 1). The laboratory tests showed high levels of serum parathyroid hormone and calcitonin, normal levels of serum calcium, phosphorus, and Vitamin D. A 24-hour urine collection found elevated levels of norepinephrine, epinephrine, and methyldopa (Table 1). Because of our patient’s familial history of MEN 2A, a genetic mutational analysis for the *RET* proto-oncogene was performed at our hospital’s medical genetics department. The test was carried out on our patient’s peripheral blood. Her genomic deoxyribonucleic acid (DNA) was amplified by using polymerase chain reaction (PCR) and oligonucleotide primers for exon 11. A direct sequencing analyses showed a *RET* (rearranged during transfection proto-oncogene) germline mutation at exon 11 (codon 634), with a substitution of tyrosine by a cysteine residue (C1900T→C (C634R).

**Table 1:** Laboratory tests results of our patient

	Results	Normal range
Parathyroid hormone (serum)	222.2 pg/mL	11–54pg/mL
Calcitonin (serum)	3130 pg/mL	˂ 6.4 pg/mL
Calcium (serum)	10.2 mg/L	8.7–10.3mg/dL
Phosphorus (serum)	3.5 mg/L	2.5–4.5 mg/dL
Vitamin D (serum)	28 ng/mL	24–40 ng/mL
Norepinephrine (urine)	345,350 nmol/24 h	90–500 nmol/24 h
Epinephrine (urine)	217,152 nmol/24 h	˂ 120 nmol/24 h
Methyldopa (urine)	9722 nmol/24 h	300–3000 nmol/24 h

*pg* picogram, *mL* milliliter, *L* liter, *nmol* nanomole, *h* hour

Our patient had undergone bilateral adrenalectomy, total thyroidectomy with cervical lymphadenectomy, and parathyroidectomy. A pathological examination confirmed the MEN 2A consisting of left medullary pheochromocytoma, right medullary composite pheochromocytoma-ganglioneuroma, medullary carcinoma of the thyroid (MCT) with lymph node metastasis, and parathyroid hyperplasia. Our patient recovered well after surgery and had no signs of disease 3 years after treatment.

### Pathological findings

**Fig. 2:**
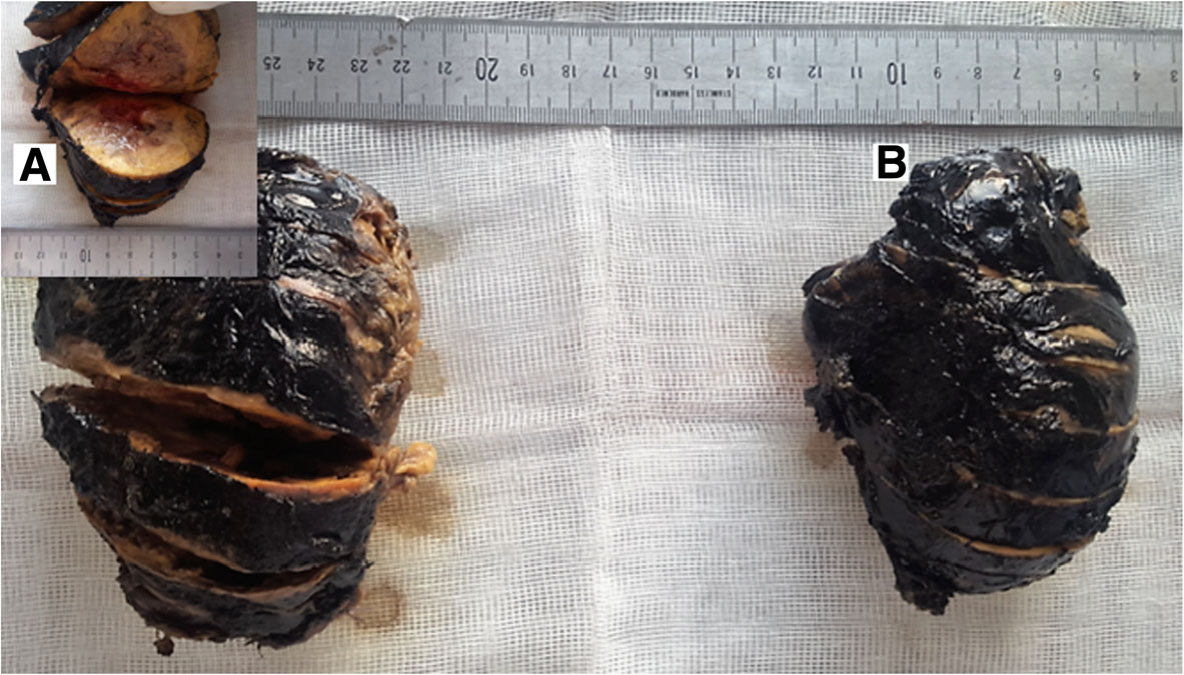
The macroscopic view of the resected adrenal glands. **a** The right adrenal gland shows a well-encapsulated tumor, the cut surface is yellow-brown, with areas of hemorrhage. **b** The left resected specimen has quite similar characteristics

Grossly, the resected right adrenal specimen measured 9 × 6.5 × 5 cm. The tumor was well-encapsulated, firm, and the cut surface was yellow-brown, with areas of hemorrhage (Fig. 2a).

The left adrenal specimen measured 13 × 8 cm. The cut surface of the tumor was light tan and yellowish, with hemorrhagic changes (Fig. 2b).

The left lobe of the resected thyroid gland measured 4.5 × 3.5 × 1.5 cm; the right lobe measured 5 × 3 × 3 cm and the isthmus measured 2 × 1.5 cm. A 1.7 × 1.2 cm nodule was found in the middle third of the left lobe; it was fleshy, grayish-white, and ill-defined. Cystic and fibrous degeneration were seen in the remaining parenchyma. The right lobe was enlarged with multinodular colloid structures with cystic degeneration.

The resected parathyroid gland measured 2.4 × 1 × 0.5 cm, with no macroscopic lesion identified.

Lymphadenectomy consisted of fibroadipocytic fragments measuring from 0.5 cm to 3 × 2.5 cm, containing several lymph nodes.

**Fig. 3:**
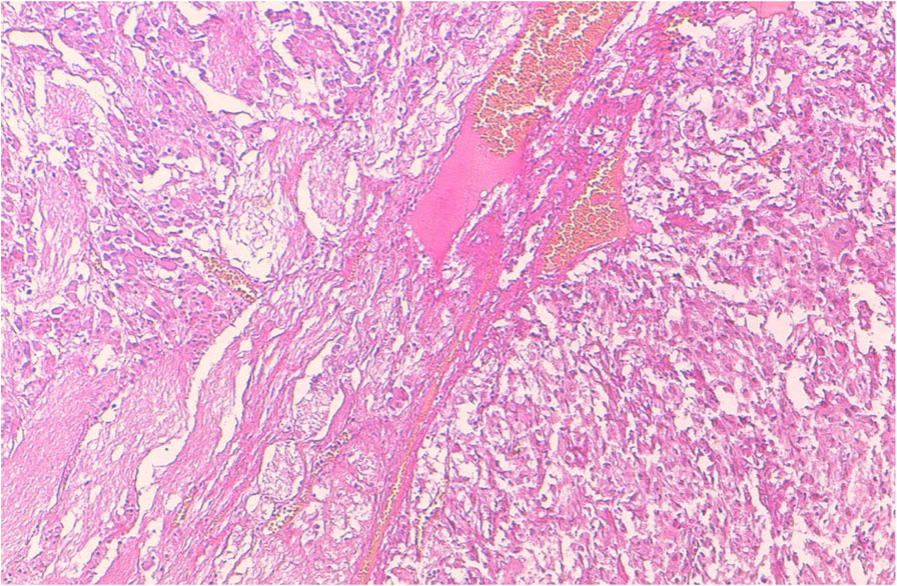
The two components of the tumors of the right adrenal gland: pheochromocytoma (*the right part of the image*) and ganglioneuroma (*the left part*), separated by dilated blood vessels (Hematoxylin-eosin-safran (HES) ×100)

**Fig. 4:**
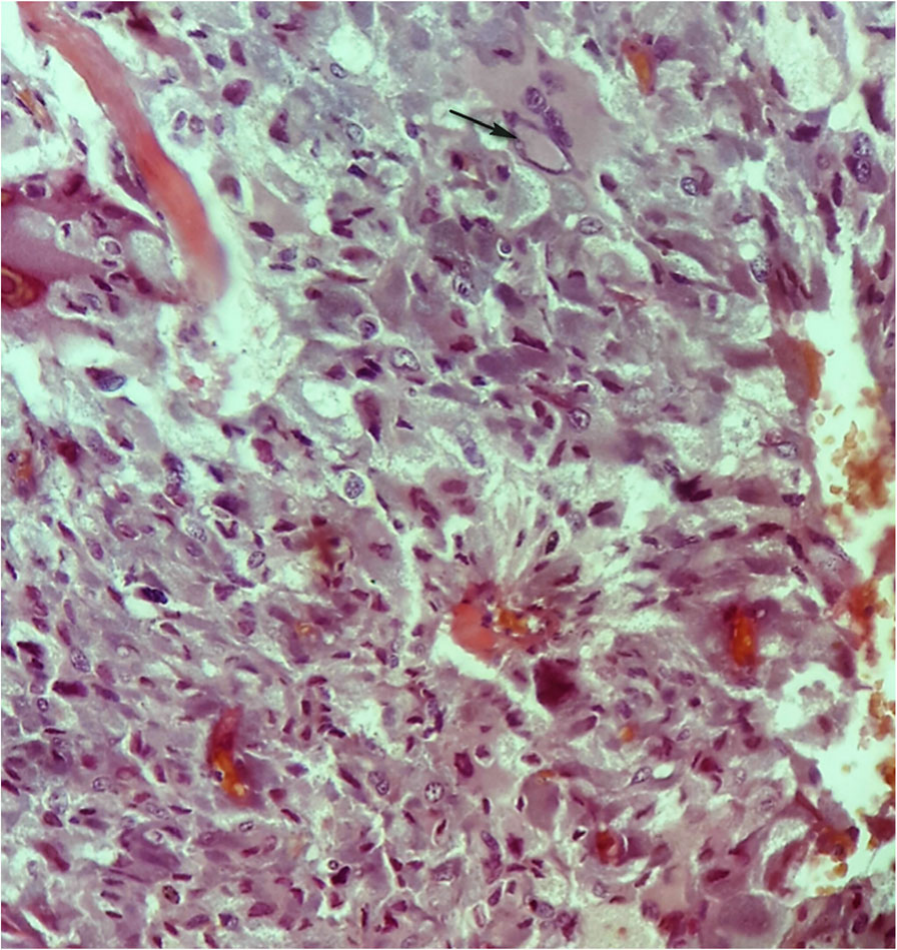
The pheochromocytoma component of the tumor shows polygonal, oval-shaped and spindle cells with amphophilic cytoplasm. The nuclei were round to spindle-shaped with inconspicuous nucleoli. A bizarre cell is seen with pseudoinclusion at the top part of the image (*black arrow*) (Hematoxylin-eosin-safran (HES) ×400)

**Fig. 5:**
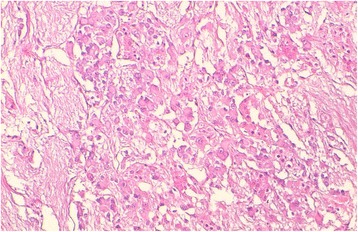
The ganglioneuroma component of the tumor shows multiple mature ganglion cells with abundant eosinophilic cytoplasm and round nuclei with conspicuous nucleoli, within a fibrillary background (Hematoxylin-eosin-safran (HES) ×200)

A histologic examination with routine hematoxylin-eosin staining (HES) of the right adrenal resected specimen showed tumor cells arranged in trabecular and anastomosing cords. The cells were polygonal, spindle or oval-shaped with a moderate amphophilic cytoplasm. The nuclei were round to spindle-shaped with inconspicuous nucleoli. Some bizarre nuclei were found with pseudoinclusion (Figs. 3 and 4). Beside this component, a second one was found consisting of mature ganglion cells with abundant eosinophilic cytoplasm and a prominent nucleolus, embedded in a thick fibrillary matrix background (Fig. 5). The two components were slightly separated by dilated blood vessels. Mitosis figures or necroses were not observed. The ganglioneuroma component represented approximately 20% of the tumor surface.

The microscopic aspect of the left adrenal resected specimen was similar to the pheochromocytoma component seen in the right adrenal specimen.

**Fig. 6:**
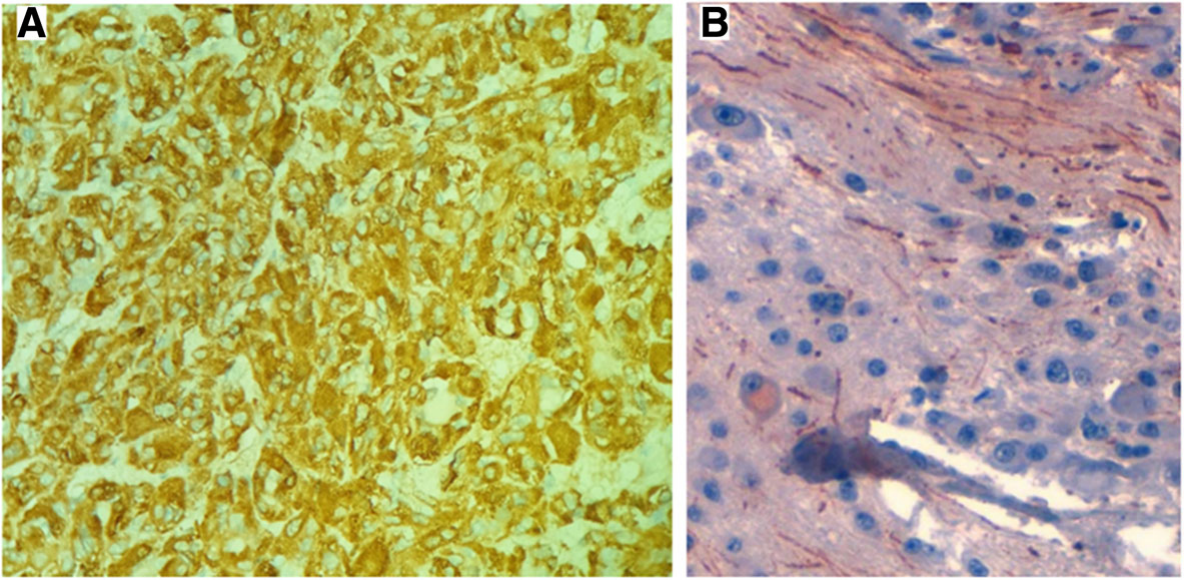
The cells of the pheochromocytoma component are strongly positive for chromogranin (**a**). The ganglion cells of the ganglioneuroma component are positive for neurofilament as well as the Schwannian fibrillary matrix (**b**) (×100)

At immunohistochemistry, the cells of the pheochromocytoma component were strongly positive for synaptophysin and chromogranin, but negative for neurofilament (Fig. 6a). The ganglion cells of the ganglioneuroma components were negative for chromogranin and stained positive for neurofilament, as well as the Schwannian fibrillary matrix (Fig. 6b).

**Fig. 7:**
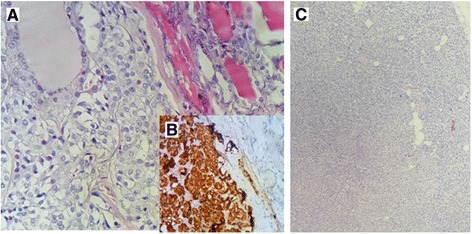
The medullary carcinoma of the thyroid shows sheets of polygonal cells with amphophilic cytoplasm and oval nuclei with granular chromatin and inconspicuous nucleoli (**a**) (HES ×200). These cells are strongly positive for chromogranin (**b**). The hyperplastic parathyroid shows a significant decrease in adipocytic lobules (**c**) (Hematoxylin-eosin-safran (HES) ×200)

Microscopically, the nodule of the left lobe of the resected thyroid gland showed sheets of polygonal cells with amphophilic cytoplasm and oval nuclei with granular chromatin and inconspicuous nucleoli. Amyloid deposits were seen as pink amorphous globules within the tumor sheets (Fig. 7a).

Tumor cells stained positive for synaptophysin and chromogranin, but negative for thyroid transcription factor 1 (TTF1) (Fig. 7b).

The histologic examination of the parathyroid gland showed hyperplastic chief cells with reduced adipose tissue (Fig. 7c).

The lymphadenectomy consisted of 26 lymph nodes with two metastatic lymph nodes from medullary carcinoma of the thyroid. The tumor was classified as pT1bN1b (AJCC 2009).

## Discussion

Composite pheochromocytoma/paragangliomas (CPC/PG) are uncommon tumors combining the histologic features of the ordinary PC/PG with those of ganglioneuroma, ganglioneuroblastoma, neuroblastoma or peripheral nerve sheath tumors [2]. Paragangliomas (PG) are tumors derived from paraganglia located along the supradiaphragmatic (parasympathetic) nerves, the pre- and paravertebral sympathetic chains or sympathetic nerve fibers of the pelvic and retroperitoneal organs. Pheochromocytoma is a paraganglioma of the adrenal gland. Cells of both adrenal medulla and paraganglia derived embryologically from neural crest cells that migrated to other organs [2, 3]. This embryologic common origin allows understanding the histogenesis of composite tumors. In fact, neural crest cells migrate to other sites and mature into melanocytes, chromaffin cells, Schwann cells or ganglion cells [2, 10]. Disorders of crest cells migration or maturation at various stages of the cellular development are thought to lead to CPC/PG [2, 5]. The possibility of chromaffin cells to undergo divergent differentiation into neuronal elements is another hypothesis to explain histogenesis of CPC/PG [11].

Macroscopically, CPC/PG have similar features of ordinary pheochromocytoma, presenting generally, as solid and firm tumors, the cut section appearing gray-white, light tan, or dusky red [10, 12]. The histologic diagnosis of composite pheochromocytoma/paraganglioma (CPC/PG) must be based both on architecture and on cell populations deriving from neural crest. Any tumor component that does not share common embryological origin with paraganglia or adrenal medulla cells should be considered as a collision tumor [2, 12]. In addition, the presence of degenerating chromaffin cells, lymphocytes, or scattered neuron-like cells, should not be misinterpreted as CPC/PG, as they are occasionally found in ordinary PC/PG [6, 12]. On microscopic examination, the two components of the tumor are admixed or separate, usually the pheochromocytoma is the predominant component [6, 7]; but the case reported by Brady *et al*. was composed of approximately equal parts of PC and PG [6]. The PC cells are arranged in syncytial-like growth pattern (Zellballen pattern), or arranged in an alveolar, diffuse, trabecular or solid pattern. Spindle cells can also be found in the tumor. Cellular pleomorphism is often present, with admixture of large and small cells. Some cells with bizarre appearance with nuclear pseudoinclusion may be observed, but this is not criteria for malignancy [12]. The stroma and the vasculature of the tumor may be prominent, causing unhabitual appearance, with diagnostic challenge [2]. In our case, the PC component showed trabecular and diffuse pattern with a rich vasculature; cells with pseudoinclusion and spindle cells have been observed. The ganglioneuroma component was slightly demarcated from the PC component by dilated blood vessels. Immunochemistry is very helpful, to rule out differential diagnosis or to distinguish easily the different components of the CPC/PG. The cells of the PC/PG component stain positive for neuroendocrine markers such as chromogranin and synaptophysin; functional tumors are positive for catecholamine-synthetizing enzymes such as thyrosine hydroxylase (TH) or phenylethanolamine N-methyltransferase (PNMT). Tumor cells have also capacity to produce a large variety of neuropeptides like vasoactive intestinal peptide (VIP), bombesin, substance P, and so on. [2, 6, 7]. The neurogenic cells are positive for neuron-specific enolase (NSE), neurofilament (NF), but show weak or focal staining for chromogranin. The sustentacular cells and Schwann cells are positive for S-100 protein [6, 12]. There is no reliable biological marker that reflects the malignant potential of CPC/PG. Comstock *et al*. reported the absence of *N-myc* amplification in four cases of CPC, and concluded that CPC does not have adverse prognostic significance conferred by the neuroblastic elements [13].

Usually, CPC/PG are functional tumors, clinical symptoms are related to the type of hormones produced by the component of the tumor. Symptoms range from palpitations, headache, hypertension, and excessive sweat. These signs are due to excessive production of epinephrine or norepinephrine. Cases presenting with watery diarrhea have been described attributable to increased production of vasoactive intestinal peptide (VIP) [12]. According to Shawa *et al*., CPC/PG are similar clinically and radiologically, and their management should be so [14].

PC/PG, ordinary or composite types, are usually associated with certain hereditary disorders: von Hippel-Lindau syndrome (vHL), multiple endocrine neoplasia syndrome type 2A and 2B (MEN 2A and 2B), NF1 and the recently described familial paraganglioma syndrome [2, 12]. Several genes are involved in the pathogenesis of PC, such as the *RET* proto-oncogene, von Hippel–Lindau disease tumor suppressor (*VHL*), neurofibromatosis type 1 tumor suppressor (*NF1*), genes encoding the succinate dehydrogenase (SDH) complex subunits, *TMEM127*, and MYC-associated factor X (*MAX*) [4].

To the best of our knowledge, only four cases of CPC/PG associated with MEN 2 have been reported [6–9] (Table 2). MEN type 2 is an autosomal-dominant disease with major components of MTC, PC, and hyperparathyroidism [7, 9]. There were two cases of MEN 2A and two cases of MEN 2B. The case reported by Matias-Guiu *et al*. was in fact a postmortem diagnosis on autopsy as the patient had died after an orthopedic surgery [7]. He had a *RET* germline mutation on the exon 11 at the codon 634, like our patient. In fact the vast majority of patients with MEN 2A carry a *RET* germline mutation on exon 10 or 11 [7, 9]. The MEN 2B represents less than 10% of the MEN 2 entity and has a more aggressive course. Patients with MEN 2B develop MTC, pheochromocytomas, developmental abnormalities, mucosal neuromas, and intestinal ganglioneuromas. They carry mostly a germline methionine-to-threonine mutation at codon 918 (M918T) in exon 16 of the *RET* proto-oncogene [9]. The case reported by Yamasaki *et al*. had a composite retroperitoneal paraganglioma with a M918T germline mutation of the *RET* proto-oncogene [9]. Patients’ age ranged from 27 years to 59 years, with a male predominance (three men for one woman). Our case was a woman aged 40 years. The adrenal medulla was the most common site of the tumors in the previously reported cases. However, the case reported by Yamasaki *et al*. had a retroperitoneal CPG. Our patient had bilateral pheochromocytomas with a CP at the right adrenal medulla. The neurogenic components of CPC/PG are, in decreasing frequency: ganglioneuroma, ganglioneuroblastoma, neuroblastoma, malignant peripheral nerve sheath tumors (MPNST), and neuroendocrine carcinoma [2].

**Table 2:** Reported cases of composite pheochromocytoma/paragangliomas associated with MEN 2A/B

Age(years)/sex	MEN type	Tumor size/weight	Organ	Neurogenic component	Outcome	Reference/country/year
34/M	2A	1.6 cm/11,8g	Left adrenal medulla	Ganglioneuroma	Uneventful postoperative course	Brady *et al*. [6]/USA/1997
49/M	2A	600g	Left adrenal medulla	Ganglioneuroblastoma	Died after orthopedic surgery (postmortem incidental finding)	Matias-Guiu *et al.* [7]/Spain/1998
27/F	2B	10 cm/124g	Left adrenal medulla	Ganglioneuroma	Tumor free for 16 months after surgery	Charfi *et al.* [8]/Tunisia/2008
59/M	2B	3 cm	Retroperitoneum	Ganglioneuroma	Metastatic disease at diagnosis	Yamasaki *et al*. [9]/Japan/2016
40/F	2A	9 cm	Right adrenal medulla	Ganglioneuroma	Tumor free for 3 years	Our case/Morocco

*MEN2A/2B* multiple endocrine neoplasia type 2A or 2B, *M* Male, *F* Female

The clinical course of CPC/PG is not clearly defined because of the rarity of these tumors. Patients usually present with clinical signs related to hormone hyperproduction, such as headache, palpitations or hypertension. It has been reported in the literature that the prognosis of composite tumors with neuroblastoma (NB), ganglioneuroblastoma (GNB) and MPNST is worse, and the clinical behavior is determined by these components [2]. The behavior of CPC/PG associated with MEN 2 seems to depend on the MCT. In fact, cases reported by Matias-Guiu *et al*. and Yamasaki *et al*., as well as our case, had metastasis from MCT [7, 9]. In general, the prognosis of patients with MEN 2 relies on the occurrence of the MCT, as the associated pheochromocytomas or other symptoms often have a benign course [15]. Recently, the American Thyroid Association (ATA) has updated its guidelines for the management of MTC [15]. In fact, the aggressiveness of MTC in MEN 2 depends on the type of *RET* proto-oncogene mutations, and a subsequent risk stratification has been suggested by the ATA. Patients with MEN 2A and C634 mutation on exon 11, like our patient, have a high risk of aggressive MTC. Patients with M918T *RET* mutation on exon 16, in patients with MEN 2B, have the highest risk. According to ATA’s guidelines, patients with a high-risk MEN 2 (ATA-H category), like our current patient, should be treated by surgery and their first-degree relatives should be offered genetic counseling and genetic testing for *RET* germline mutations [15]. In fact, such management has been proposed to our patient’s relatives but we do not know if they have had it done elsewhere, as they have not been screened at our center. The sister of our patient had also undergone surgery for MEN 2A years before; unfortunately, her sister (our current patient) had not been screened until she developed the complete disease syndrome with a locally advanced MTC.

## Conclusions

CPC/PGs are very rare tumors and are supposed to have similar clinical behavior of ordinary pheochromocytoma/paraganglioma. The association with MEN 2 is uncommon. We have described here a case of a patient with CPC associated with MEN 2A, together with a review of the literature. Less than 100 cases of CPC/PG have been reported, emphasizing the need for further studies in order to know more about the epidemiological, diagnostic, and prognostic aspects of these rare tumors.

## Abbreviations

ATA: American Thyroid Association
CPC/PG: Composite pheochromocytoma-paraganglioma
DNA: Deoxyribonucleic acid
GN: Ganglioneuroma
GNB: Ganglioneuroblastoma
HES: Hematoxylin-eosin Saffron
MCT: Medullary carcinoma of the thyroid
MEN 2A/B: Multiple endocrine neoplasia syndrome type 2A/type 2B
MPNST: Malignant peripheral nerve sheath tumors
NF1: Neurofibromatosis type 1
NSE: Neuron-specific enolase
PC: Pheochromocytoma
PCR: Polymerase chain reaction
PG: Paraganglioma
RET: Rearranged during transfection proto-oncogene
vHL: von Hippel Lindau syndrome
VIP: Vasoactive intestinal peptide
